# Author Correction: Investigation of a UK biobank cohort reveals causal associations of self-reported walking pace with telomere length

**DOI:** 10.1038/s42003-022-03459-w

**Published:** 2022-05-19

**Authors:** Paddy C. Dempsey, Crispin Musicha, Alex V. Rowlands, Melanie Davies, Kamlesh Khunti, Cameron Razieh, Iain Timmins, Francesco Zaccardi, Veryan Codd, Christopher P. Nelson, Tom Yates, Nilesh J. Samani

**Affiliations:** 1 Diabetes Research Centre, University of Leicester, Leicester General Hospital, Leicester, UK; 2 NIHR Leicester Biomedical Research Centre, Leicester General and Glenfield Hospitals, Leicester, UK; 3 Baker Heart and Diabetes Institute, Melbourne, VIC Australia; 4 MRC Epidemiology Unit, University of Cambridge, Cambridge, UK; 5 Department of Health Sciences, University of Leicester, Leicester, UK; 6 Leicester Diabetes Centre, Leicester General Hospital, University Hospitals of Leicester NHS Trust, Leicester, UK; 7 NIHR Collaboration for Leadership in Applied Health Research and Care—East Midlands, University as Leicester, Leicester, UK; 8 Leicester Real World Evidence Unit, University of Leicester, Leicester General Hospital, Leicester, UK; 9 Department of Cardiovascular Sciences, University of Leicester, Leicester, UK

**Keywords:** Predictive markers, Risk factors, Biomarkers

Correction to: *Communications Biology* 10.1038/s42003-022-03323-x, published online 20 April 2022.

In this article, some numbers in Table 1 were incorrect. This has now been corrected.

