# Supplementary material for: Investigation of a UK biobank cohort reveals causal associations of self-reported walking pace with telomere length
Source: Commun Biol. 2022 Apr 20;5:381. doi: 10.1038/s42003-022-03323-x (PMC9021230; doi:10.1038/s42003-022-03323-x)
Supplement: Supplementary file 2 — Supplementary Information [file 42003_2022_3323_MOESM2_ESM.pdf]

Supplementary Material

Supplementary Table 1. Descriptive characteristics at baseline of the accelerometer analytic sample, and by self-reported walking pace.

Characteristics	Total sample	Slow	Average/steady	Brisk
	N=86,002 *	N=3,934	N=41,305	N=40,595
Age (years), mean (SD)	56.16 (7.81)	58.28 (7.29)	56.85 (7.73)	55.24 (7.84)
Female gender, n (%)	48,212 (56.1%)	2,343 (59.6%)	23,394 (56.6%)	22,373 (55.1%)
White ethnicity, n (%)	83,491 (97.1%)	3,737 (95.0%)	39,892 (96.6%)	39,708 (97.8%)
**Highest educational level achieved, n (%)**				
No qualification	7,057 (8.2%)	678 (17.2%)	4,071 (9.9%)	2,277 (5.6%)
Any other qualification	41,492 (48.2%)	2,051 (52.1%)	21,097 (51.1%)	18,268 (45.0%)
Degree level or above	37,453 (43.5%)	1,205 (30.6%)	16,137 (39.1%)	20,050 (49.4%)
Townsend indicator of multiple deprivation, median (IQR)	-2.46 (-3.83--0.23)	-1.74 (-3.40-1.26)	-2.46 (-3.82--0.23)	-2.53 (-3.86--0.35)
In employment, n (%)	53,183 (61.8%)	1,584 (40.3%)	24,308 (58.9%)	27,244 (67.1%)
**Cigarette smoking, n (%)**				
Never	49,115 (57.1%)	1,831 (46.5%)	22,869 (55.4%)	24,330 (59.9%)
Previous	30,967 (36.0%)	1,672 (42.5%)	15,448 (37.4%)	13,788 (34.0%)
Current	5,920 (6.9%)	431 (11.0%)	2,988 (7.2%)	2,477 (6.1%)
**Alcohol consumption, n (%)**				
Never or previous	4,792 (5.6%)	498 (12.7%)	2,373 (5.7%)	1,886 (4.6%)
< Twice a week	38,907 (45.2%)	2,113 (53.7%)	19,563 (47.4%)	17,139 (42.2%)
At least three times a week	42,303 (49.2%)	1,323 (33.6%)	19,369 (46.9%)	21,570 (53.1%)
**Added salt intake, n (%)**				
Never/rarely	51,602 (60.0%)	2,069 (52.6%)	24,127 (58.4%)	25,320 (62.4%)
Sometimes or more frequent	34,400 (40.0%)	1,865 (47.4%)	17,178 (41.6%)	15,275 (37.6%)
**Oily fish consumption, n (%)**				
More than once a week	48,605 (56.5%)	2,045 (52.0%)	22,584 (54.7%)	23,879 (58.8%)
Fruit and vegetable intake score, median (IQR)	2.00 (1.00-2.00)	1.00 (1.00-2.00)	2.00 (1.00-2.00)	2.00 (1.00-3.00)
Weekly frequency of red or processed meat intake, median (IQR)	0.75 (0.50-1.13)	0.88 (0.50-1.25)	0.75 (0.50-1.25)	0.75 (0.50-1.13)
**Mean sleep duration, n (%)**				
<7 hours/day	18,703 (21.7%)	1,129 (28.7%)	8,961 (21.7%)	8,561 (21.1%)
7-8 hours/day	61,878 (71.9%)	2,252 (57.2%)	29,465 (71.3%)	30,087 (74.1%)
>8 hours/day	5,421 (6.3%)	553 (14.1%)	2,879 (7.0%)	1,947 (4.8%)
**Body mass index, n (%)**				
Normal weight (<25 kg/m2)	34,035 (39.6%)	631 (16.0%)	12,920 (31.3%)	20,441 (50.4%)
Overweight (25-30 kg/m2)	35,449 (41.2%)	1,249 (31.7%)	18,175 (44.0%)	15,976 (39.4%)

Obese (≥30 kg/m2)	16,518 (19.2%)	2,054 (52.2%)	10,210 (24.7%)	4,178 (10.3%)
Current prescription of blood pressure or cholesterol medicine, n (%)	19,627 (22.8%)	1,793 (45.6%)	10,781 (26.1%)	6,965 (17.2%)
Diagnosis of diabetes or insulin prescription, n (%)	2,952 (3.4%)	466 (11.8%)	1,627 (3.9%)	826 (2.0%)
Previous diagnosis of cardiovascular disease, n (%)	4,187 (4.9%)	638 (16.2%)	2,228 (5.4%)	1,292 (3.2%)
Previous diagnosis of cancer, n (%)	7,085 (8.2%)	431 (11.0%)	3,560 (8.6%)	3,075 (7.6%)
Mobility limitation, n (%)	30,480 (35.4%)	2,990 (76.0%)	15,700 (38.0%)	11,643 (28.7%)
Total physical activity (m*g*), mean (SD)	28.32 (8.41)	22.65 (7.35)	27.09 (7.78)	30.15 (8.64)
Intensity gradient, mean (SD)	-2.55 (0.19)	-2.67 (0.20)	-2.57 (0.19)	-2.50 (0.19)
**Self-reported walking pace, n (%) ***				
Slow	3,934 (4.6%)	-	-	-
Average/steady	41,305 (48.0%)	-	-	-
Brisk	40,595 (47.2%)	-	-	-
Missing	168 (0.2%)	-	-	-
Total white blood cell (Leukocyte) count (10^9 cells/Litre), median (IQR)	6.49 (5.51-7.61)	7.20 (6.10-8.50)	6.60 (5.62-7.77)	6.30 (5.40-7.40)
Telomere length (z-score), mean (SD)	0.000 (1.000)	-0.110 (0.985)	-0.026 (1.004)	0.037 (0.995)

* note: n=168 did not have walking pace data in the total (analytic) accelerometer sample (n=86,002) used in subsequent analyses with LTL.

Townsend score, a composite area-level measure of deprivation based on unemployment, non-car ownership, non-home ownership, and household overcrowding; a higher score indicates higher deprivation.

Supplementary Table 2. Mendelian randomisation between self-reported walking pace and LTL

Part 1: LTL on walking pace (118/130 SNPs for LTL)

	Unadjusted walking pace I^2 = 55.2%			Walking pace adjusted for BMI I^2 = 37.7%		
	**Beta* (95% CI)**	**P-value**	**Egger - p**	**Beta* (95% CI)**	**P-value**	**Egger - p**
**MR-IVW**	0.004 (-0.011, 0.019)	0.594	0.689	0.005 (-0.007, 0.016)	0.451	0.750
**MR-WM**	0.003 (-0.013, 0.019)	0.685		0.001 (-0.014, 0.016)	0.859	
**MR-RAPS**	0.004 (-0.006, 0.014)	0.419		0.005 (-0.005, 0.014)	0.336	

Part 2: Walking pace on LTL (67 SNPs for walking pace)

	Unadjusted walking pace I^2 = 61.1%			Walking pace adjusted for BMI I^2 = 63.6%		
	**Beta† (95% CI)**	**P-value**	**Egger - p**	**Beta† (95% CI)**	**P-value**	**Egger - p**
**MR-IVW**	0.186 (0.088, 0.283)	1.99E-04	0.685	0.209 (0.069, 0.348)	0.003	0.094
**MR-WM**	0.107 (0.011, 0.204)	0.030		0.108 (-0.023, 0.239)	0.105	
**MR-RAPS**	0.198 (0.136, 0.260)	3.51E-10		0.236 (0.151, 0.322)	6.36E-08	

Where MR-IVW is the inverse-variance weighted MR which was used as the primary MR method, with MR-WM as the weighted-median MR and MR-RAPS as the robust adjusted profile score MR which were both included as sensitivity analyses. Beta* is the estimated unit difference in walking pace per 1SD increase in LTL, and Beta† is the SD change in LTL per 1 unit difference in walking pace, where a 1 unit increase in self-rated walking pace represents a change in category from slow to steady/average, or from steady/average to brisk pace.

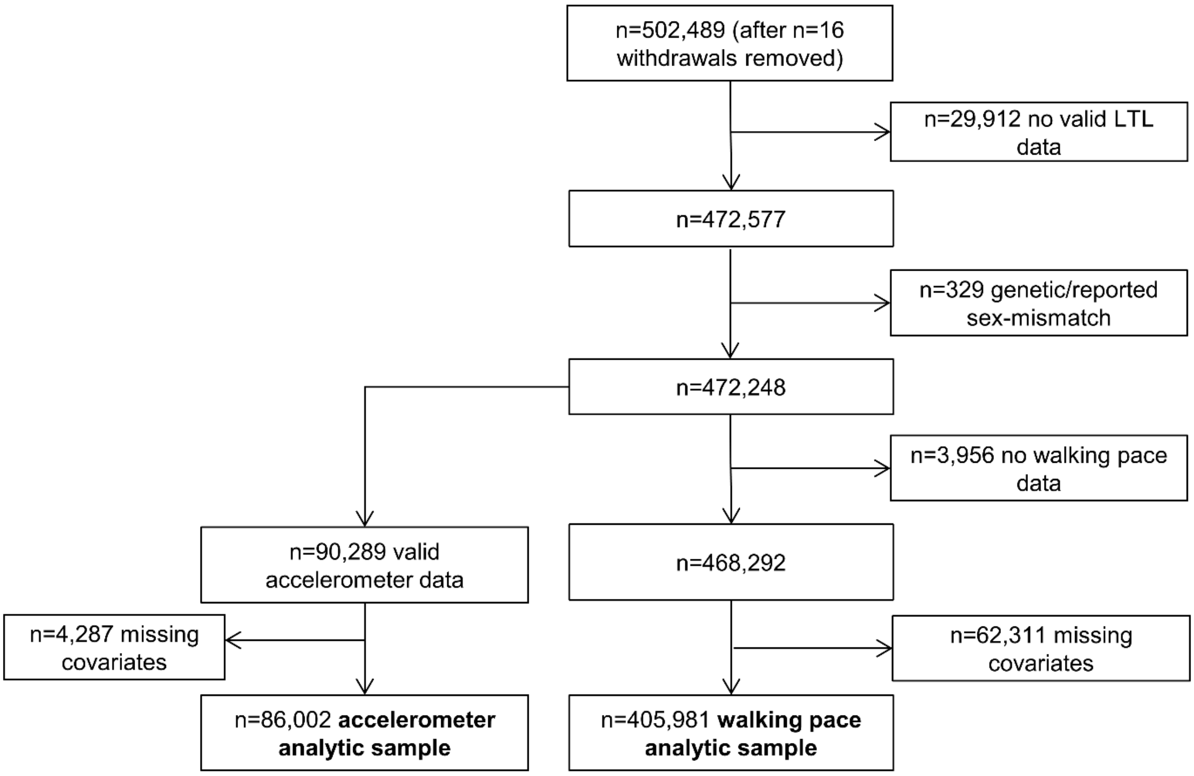

Supplementary Figure 1. Flowchart of participant exclusions.

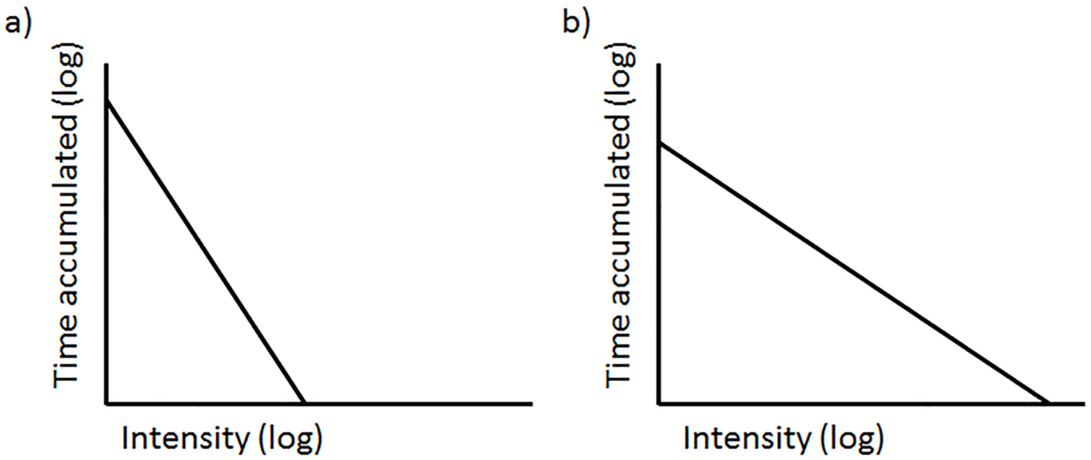

Supplementary Figure 2. Illustration of the intensity gradient metric.

a) a steeper, more negative (lower) gradient with a higher constant (y-intercept) showing a steep drop in time accumulated with increasing intensity (left)—a ‘poorer’ intensity profile.

b) a shallower, less negative (higher) gradient with a lower constant (y-intercept) showing more time spread across the intensity range (right)—a ‘better’ intensity profile.

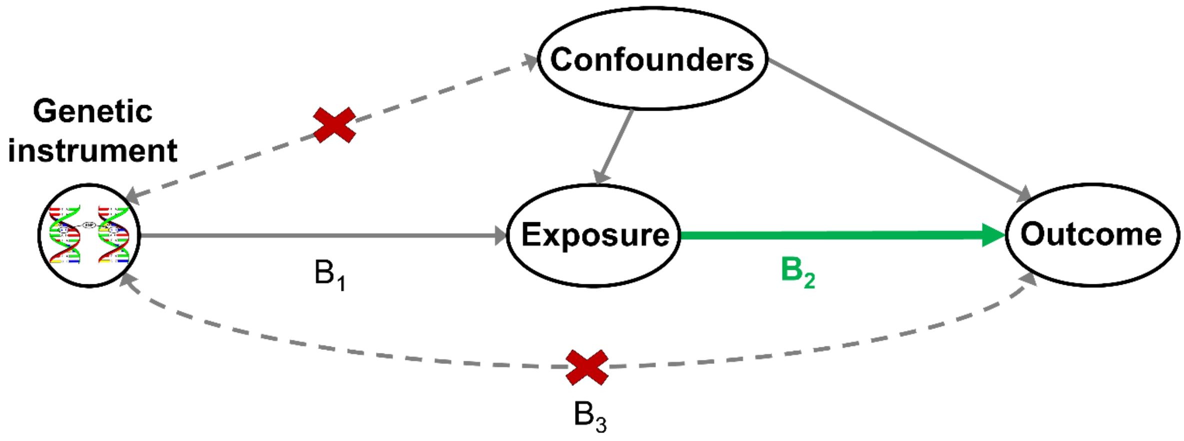

Supplementary Figure 3. Simplified causal diagram illustrating Mendelian randomization and its assumptions.

Solid pathway lines are theorized to exist; dashed pathway lines are theorized to be nonsignificant according to model assumptions. B_2 indicates the estimated causal relationship ($B_2 = B_1/B_3$). B_1 and B_3 indicate the estimated direct effects of a genetic variant on the exposure (e.g. walking pace) and outcome (e.g. LTL).

To be valid instrumental variables for the causal association of walking pace on LTL, or vice versa, genetic variants (SNPs) must be: 1) associated with the exposure of interest (e.g. walking pace), 2) independent of factors that confound the association of the exposure and outcome, and 3) associated with the outcome (e.g. LTL) exclusively through their effects on the exposure. If these assumptions are satisfied, the selected SNPs are valid instrumental variables, and their association with disease can be interpreted as a causal effect of the exposure.
