# Supplementary material for: Investigation of a UK biobank cohort reveals causal associations of self-reported walking pace with telomere length
Source: Commun Biol. 2022 Apr 20;5:381. doi: 10.1038/s42003-022-03323-x (PMC9021230; doi:10.1038/s42003-022-03323-x)
Supplement: Supplementary file 3 — Reporting Summary [file 42003_2022_3323_MOESM3_ESM.pdf]

nature portfolio

Corresponding author(s): Dr Paddy Dempsey

Last updated by author(s): Mar 18, 2022

Reporting Summary

Nature Portfolio wishes to improve the reproducibility of the work that we publish. This form provides structure for consistency and transparency in reporting. For further information on Nature Portfolio policies, see our Editorial Policies and the Editorial Policy Checklist.

Statistics

For all statistical analyses, confirm that the following items are present in the figure legend, table legend, main text, or Methods section.

n/a	Confirmed	
☐	☒	The exact sample size (*n*) for each experimental group/condition, given as a discrete number and unit of measurement
☐	☒	A statement on whether measurements were taken from distinct samples or whether the same sample was measured repeatedly
☐	☒	The statistical test(s) used AND whether they are one- or two-sided<br>*Only common tests should be described solely by name; describe more complex techniques in the Methods section.*
☐	☒	A description of all covariates tested
☐	☒	A description of any assumptions or corrections, such as tests of normality and adjustment for multiple comparisons
☐	☒	A full description of the statistical parameters including central tendency (e.g. means) or other basic estimates (e.g. regression coefficient) AND variation (e.g. standard deviation) or associated estimates of uncertainty (e.g. confidence intervals)
☐	☒	For null hypothesis testing, the test statistic (e.g. *F*, *t*, *r*) with confidence intervals, effect sizes, degrees of freedom and *P* value noted<br>*Give P values as exact values whenever suitable.*
☒	☐	For Bayesian analysis, information on the choice of priors and Markov chain Monte Carlo settings
☒	☐	For hierarchical and complex designs, identification of the appropriate level for tests and full reporting of outcomes
☐	☒	Estimates of effect sizes (e.g. Cohen's *d*, Pearson's *r*), indicating how they were calculated

Our web collection on statistics for biologists contains articles on many of the points above.

Software and code

Policy information about availability of computer code

Data collection	Data collection was undertaken elsewhere and this is clearly stated in the manuscript.
Data analysis	All analyses were undertaken in Stata v16 (StataCorp LLC, College Station, TX) as stated in the manuscript, as well as custom software BOLT-LMMv2.3.2, PLINKvl.9, FUMA, KING, LDSCvl.0 1, ORDINALGWAS.JL, PRSICEv2.2.3

For manuscripts utilizing custom algorithms or software that are central to the research but not yet described in published literature, software must be made available to editors and reviewers. We strongly encourage code deposition in a community repository (e.g. GitHub). See the Nature Portfolio guidelines for submitting code & software for further information.

Data

Policy information about availability of data

All manuscripts must include a data availability statement. This statement should provide the following information, where applicable:

- Accession codes, unique identifiers, or web links for publicly available datasets
- A description of any restrictions on data availability
- For clinical datasets or third party data, please ensure that the statement adheres to our policy

The UK Biobank data that support the findings of this study can be accessed by researchers on application (https://www.ukbiobank.ac.uk/register-apply/). Variables derived specifically for this study will be returned along with the code to the UK Biobank for future applicants to request.

Field-specific reporting

Please select the one below that is the best fit for your research. If you are not sure, read the appropriate sections before making your selection.

☐ Life sciences ☒ Behavioural & social sciences ☐ Ecological, evolutionary & environmental sciences

For a reference copy of the document with all sections, see nature.com/documents/nr-reporting-summary-flat.pdf

Behavioural & social sciences study design

All studies must disclose on these points even when the disclosure is negative.

Study description	A cross-sectional observational population-based study of quantitative UK Biobank data. Also includes Mendelian Randomisation analyses.
Research sample	For analyses presented in this paper, we included all participants with LTL measured from the UK Biobank baseline sample, where there was no mismatch in self-reported and genetic sex (n=472,248). All participants were initially invited to the main study if their National Health Service contact details had them registered within a reasonable travelling distance of an assessment centre.
Sampling strategy	A stratified approach was taken to the initial invites for the main UK Biobank Study with over-sampling of some age, gender and deprivation sub-groups. Participation was voluntary and the response rate was 5.5%. Invites to the sub-study were sent randomly, although this was within the subset of participants that provided a valid email address. The response rate was 44.8%.
Data collection	The main exposure measure was self-reported walking pace using touchscreen self-administered questionnaire. The majority of the covariate data including demographic, other lifestyle, and health status were collected via a touchscreen self-administered questionnaire completed at assessment centres. Anthropometric measures were also obtained at these visits. Health status data were supplemented using linked hospital episode statistics.
Timing	Exposure and covariate data from the UK Biobank baseline sample (2006-2010) were used for these analyses<br><br>The physical activity accelerometry measurement occurred between 2013 and 2016. This was a median of 5.7 years after the baseline recruitment assessment centre visits that occurred between 2006 and 2010. A minority of participants undertook follow-up assessment centre visits in the period 2008 to 2018. Covariate data at baseline were used.
Data exclusions	For analyses presented in this paper, we included all participants with LTL measured from the UK Biobank baseline sample, where there was no mismatch in self-reported and genetic sex (n=472,248). Exclusions were also made for missing walking pace or covariate data, or for missing accelerometer data in the subset analysis (Supplementary Figure S1).
Non-participation	The response rates for the main UK Biobank Study and accelerometer sub-study were 5.5% and 44.8% respectively, as described in the sampling strategy.
Randomization	This was an observational study and so there was no randomisation. Covariate selection was made a priori, based on previous literature with the aim of causally inferring the relationship between physical activity and mortality. Based on our assumptions of how the covariates influence the relationship under study, we grouped covariates into those potentially on the causal pathway, and those not on the causal pathway. We performed statistical models that progressively adjusted for different groups of covariates.

Reporting for specific materials, systems and methods

We require information from authors about some types of materials, experimental systems and methods used in many studies. Here, indicate whether each material, system or method listed is relevant to your study. If you are not sure if a list item applies to your research, read the appropriate section before selecting a response.

Materials & experimental systems

n/a	Involved in the study
☒	☐ Antibodies
☒	☐ Eukaryotic cell lines
☒	☐ Palaeontology and archaeology
☒	☐ Animals and other organisms
☐	☒ Human research participants
☒	☐ Clinical data
☒	☐ Dual use research of concern

Methods

n/a	Involved in the study
☒	☐ ChIP-seq
☒	☐ Flow cytometry
☒	☐ MRI-based neuroimaging

Human research participants

Policy information about studies involving human research participants

Population characteristics	The UK Biobank consists of UK residents aged between 40 and 69 at recruitment from the year 2010, of predominantly white European ancestry. In the study sample the mean age was 56.8 and proportion of males was 45.7%.
Recruitment	The above section "Sampling strategy" describes the participant recruitment. The voluntary nature of participation, the low response rate, and the further selection pressure from the requirement to provide a valid email address means that this sample is not representative of the UK population. However, as our discussion notes, the median value for physical activity energy expenditure in this sample (a main exposure measure) is comparable to nationally representative age-specific estimates.<br><br>Participants were invited by letter and self-selected into the study. Research has demonstrated lower rates of some diseases, such as type 2 diabetes, in the UK Biobank compared to the general population, and higher socio-economic category on average. This may create selection bias in this study if direct genetic effects of walking pace are confounded by common causes of study participation and walking pace. We conditioned analyses on BMI to allow for this possibility.
Ethics oversight	The UK Biobank study has ethics approval from the North West National Research Ethics Committee (REC reference 11/NW/0274).

Note that full information on the approval of the study protocol must also be provided in the manuscript.
